# Consideration of sense of coherence in a structured communication approach with stage IV lung cancer patients and their informal caregivers: a qualitative interview study

**DOI:** 10.1007/s00520-020-05724-2

**Published:** 2020-09-03

**Authors:** Katja Krug, Jasmin Bossert, Lydia Stooß, Anja Siegle, Matthias Villalobos, Laura Hagelskamp, Corinna Jung, Michael Thomas, Michel Wensing

**Affiliations:** 1 Department of General Practice and Health Services Research, Heidelberg University Hospital, Im Neuenheimer Feld 130.3, 69120 Heidelberg, Germany; 2 Thoraxklinik Heidelberg, Department of Thoracic Oncology, Heidelberg University Hospital, Röntgenstraße 1, 69126 Heidelberg, Germany; 3 Medical School Berlin, Calandrellistr. 1-9, 12247 Berlin, Germany

**Keywords:** Communication, Lung neoplasms, Oncology, Palliative care, Patient participation, Sense of coherence

## Abstract

**Objective:**

Salutogenetic aspects are valuable for consideration in patient-centred care of advanced oncological diseases with a limited life expectancy. The Milestone Communication Approach (MCA), involving physician-nurse tandems, addresses specific challenges and needs over the disease trajectory of patients with stage IV lung cancer and their informal caregivers. This study aims to explore patients’ and informal caregivers’ salutogenetic experiences with the MCA concept.

**Methods:**

This qualitative study used face-to-face semi-structured interviews with patients and informal caregivers. All generated data were audio-recorded, pseudonymised and transcribed verbatim. Data were structured using Qualitative Content Analysis. The material was coded deductively into themes related to the components of sense of coherence (Aaron Antonovsky) and emerging sub-themes. All data was managed and organised in MAXQDA.

**Results:**

In 25 interviews, sense of coherence was referred to with all three components: “Comprehensibility” was supported by information conveyed suitably for the patients; “meaningfulness” was addressed as accepting the situation; and “manageability” led to advance care planning the patients were comfortable with. Patients and informal caregivers experienced the interprofessional tandem as an added value for patient care.

**Conclusions:**

Participants appreciate the MCA in its support for coping with a life-limiting disease. Considering salutogenetic aspects facilitates prognostic awareness and advance care planning. Nevertheless, individual needs of patients and informal caregivers require an individualised application of the MCA.

## Background

The diagnosis of cancer is potentially life-threatening for those affected. Improvement in treatment options and survival rates affects only subgroups of patients; still, the vast majority suffer from a high psychological burden, which can lead to reduced quality of life and distress. Anxious thoughts about the course of the disease are among the stress-related symptoms experienced by cancer patients [1]. Unaddressed psychological problems in clinical practice can have a negative impact on therapy outcome [2]. Therefore, considering psychosocial consequences of the disease gains in importance.

One approach for taking psychological determinants into account and to support mental regulation is the inclusion of the salutogenetic aspect “sense of coherence” (SOC) as described by Antonovsky [3]. People with a high degree of SOC are more willing to use the available internal and external resources to meet the demands of life and thus maintain a higher well-being [4].

SOC is understood as an individualised way of being, thinking and acting, which is connected with an inner trust that makes a person identify, use, apply and reuse available resources. SOC consists of three components: comprehensibility, meaningfulness, and manageability. Comprehensibility refers to the cognitive functions of an individual. It is a measure of the ability to perceive incoming information as structured and coherent. Meaningfulness refers to the ability of an individual to classify and understand the significance of events and to perceive them as a challenge. Manageability manifests itself in the conviction of the individual about their ability to cope with difficult situations, an active and effective way to influence one’s own life situation and draw conclusions from past experience [5]. Therefore, the concept of SOC could be helpful for oncological patients to understand how to deal with the situation [6].

SOC gains particular relevance in oncological diseases if the diagnosis is advanced and associated with a limited life expectancy. Living with cancer is a stressful situation not only for patients but also for informal caregivers. Families living with cancer experience symptoms of depression and anxiety especially during the palliative phase [7], when they are dependent on external support to deal with this situation. To address specific needs of patients with stage IV lung cancer and their informal caregivers, a communication approach was developed and implemented in oncology care, focused on the challenges and needs over the disease trajectory (Heidelberg Milestone Communication Approach, MCA) [8]. Stage IV lung cancer was chosen for its foreseeable disease trajectory with a limited prognosis of less than 12 months.

The MCA aims to provide patient-centred communication by an interprofessional tandem (nurse, physician) which includes psychological support for patients and informal caregivers. At an advanced stage of lung cancer, patients and informal caregivers have to deal with receiving bad news at short intervals. Topics like advance care planning (ACP) and prognostic awareness (PA) have become essential in advanced cancer stages. The MCA purposes to apply standardised communication steps through different milestone conversations (MCs) with additional follow-up telephone calls at turning points in treatment to support patients and informal caregivers in the early integration of palliative care. In this context, patient preferences are considered in order to cope with psychological burden [9]. The MCA implicitly addresses the concept of SOC, since effective coping with depression and anxiety increases comprehensibility, meaningfulness and manageability.

Against this background, this study aims to explore to which extent SOC is considered within the MCA from the perspective of patients and informal caregivers.

## Methods

### Study design

An interview study was conducted as part of a larger evaluation study [9] using a semi-structured interview guide. The interview guide was developed on the basis of a literature review and in accordance with the MCA project. Interview questions were oriented towards eliciting open-ended responses to acquire specific information on experiences with the MCA. After initially asking for the thoughts coming spontaneously to mind when remembering the MCs, the following questions focused on the experiences with the interprofessional tandem, contents of the MCs with a specific focus on decision-making and involving informal caregivers, experiences with follow-up phone calls and perceived advantages and disadvantages of the MCA. The interview guide was pre-tested with one patient and one informal caregiver to ensure that all questions were comprehensible.

### Setting

The study was conducted at the outpatient Department of Thoracic Oncology at the University Hospital Heidelberg, Germany. This hospital is a comprehensive cancer centre with a large catchment area, focused on thoracic diseases including lung cancer.

### Participants

Participating patients of the larger evaluation study were randomised into receiving either MCA or standard oncology care. Patients designated an informal caregiver (family member, significant other) who was also invited to participate. All patients and informal caregivers randomised into receiving the intervention MCA were consecutively invited to participate in semi-structured interviews after having had at least two encounters within the MCA. They met the inclusion criteria defined by the MCA study (newly diagnosed stage IV lung cancer, limited prognosis (< 12 months median), sufficient command of German, capable to fill in questionnaires of the main study, ≥ 18 years old).

### Data collection

Data were collected between September 2018 and April 2019. Face-to-face interviews were conducted additionally to regular appointments at the clinic thus complying with patients’ wishes to minimise efforts for participants. Patients and informal caregivers were interviewed separately and in a quiet room on the ward by a healthcare researcher and nurse (JB), who was not involved in patient care. All interviews were digitally recorded and transcribed verbatim. The transcripts were compared with the digital recordings to correct any inaccuracies. Data were collected until saturation was reached.

### Data analysis

Data were analysed according to Qualitative Content Analysis to structure collected data into themes and sub-themes [10]. Within this approach, two female researchers with a background in health services research and nursing (JB) and in interprofessional healthcare and radiography (LS) summarised the content deleting all expletives and repetitions. Then, the material was coded line-by-line deductively into the themes “comprehensibility”, “manageability” and “meaningfulness” as the core components of SOC. Sub-themes used in the analysis are deductively oriented towards the “Model of Communication to Integrate Early Palliative Care in Thoracic Oncology” [11]. For comprehensibility, this included language simplification and illustration of communication; portioning of information in the information process; structuring support in the treatment process; assessment support regarding limited prognosis; subsequent confirmation of understanding and decision; information support; elaboration of the treatment plausibility; and support for consideration, prioritisation and decision-making. Manageability subsumes dealing with burdensome problems concerning the disease situation, development of the social support network, and anticipatory coping advice in the context of the disease situation for palliative lung cancer patients. Meaningfulness includes practical advice on integrating private life into the palliative treatment concept, practical advice on how to keep social functions (within and outside the family), and desired equal participation in internal decision-making processes.

All interviews were analysed with this approach by both researchers to increase the reliability of the coding. The analyses were compared and the coded topics were modified where necessary. Moreover, all interviews were extensively discussed between the two researchers in order to ensure trustworthiness of the coding. Disagreements were resolved in discussions with a third researcher (KK; background in health services research and psychology).

All qualitative data were managed and analysed using MAXQDA 12 (VERBI Software GmbH, Berlin). Quotes presented as examples in this article have been translated into English with due diligence and slightly adapted to maintain meaning.

## Results

Thirteen interviews with patients (mean duration: 22 min; range: 8–54 min) and 12 interviews with informal caregivers (mean duration 20 min; range: 13–35 min) were conducted. Among the patients were 9 women and 4 men. The mean age of the patients was 65.5 years (range: 45–78 years). Age and gender were not recorded for informal caregivers.

The analysis highlighted interdependence of the SOC components as main themes in the context of the MCA.

### Theme 1: comprehensibility

Comprehensibility (the ability to receive and process relevant information) was emphasised by participants in the sub-themes “Language simplification and illustration of communication”, “Portioning of information in the information process”, “Structuring support in the treatment process”, “Assessment support regarding limited prognosis”, and “Subsequent confirmation of understanding and decision”. The remaining sub-themes “Information support”, “Elaboration of the treatment plausibility”, and “Support for consideration, prioritisation and decision-making” as important preconditions for decision-making were only occasionally mentioned in the interviews.

The sub-theme “Language simplification and illustration of communication” showed that the nurses were an important component of communication, as they often translated medical terms used by physicians in the MCs. The translation served to improve understanding between physicians, patients (*P*) and informal caregivers (*IC*).

“If it was about medical terms or something like that, [the nurse] was then an additional help (...) where you then reinterpret something which is not true at all and that is then mostly clarified with one sentence” (P17).

The sub-theme “Structuring support in the treatment process” demonstrated a further role of the nurses. Participants related this sub-theme primarily to organisational aspects. In this context, the nurse was perceived as a support when dealing with internal administrative matters, such as arranging appointments, issuing prescriptions and organizing taxi services.

“She [nurse], arranged a few things last week in this chaos of appointments for me (...) that I could not have arranged at all (...) otherwise all the appointments last week would have been cancelled.” (P05).

“They [nurses] arranged everything.” (IC31).

Statements on the sub-theme “Assessment support regarding limited prognosis” indicated that the topic of PA is considered individually for each patient in the context of MCs. While some patients did not want to talk about limited prognosis, others expected clear information from the time of diagnosis.

“I think they [interprofessional tandem] also notice [what] I don’t want to know. I’ll just put it in simple terms: a period of time left… I don’t want to know and I don’t want to hear that.” (P02).

“And then I thought, what are my life expectations? (...) Or how does that stuff [chemotherapy] work there at all and affect me? And they [interprofessional tandem] explained that to me.” (P01).

All participants were positive about the follow-up calls described by the sub-theme “Subsequent confirmation of understanding and decision”. Follow-up calls were perceived as security and comprehensive care beyond the clinical context. Individual concerns can be discussed, thus increasing well-being.

“I thought it was fantastic that there are people here in Heidelberg who care about us and call us. I’ve never experienced anything like that before.” (IC01).

“I briefly explained to her [nurse] what I had received. And then, she said: “We have already seen and heard that several times, so we have to see if we can find an alternative for you” (...) and she pointed it out again today in a conversation with the doctor.” (P02)

### Theme 2: meaningfulness

The theme “meaningfulness” included three sub-themes: “Practical advice on integrating private life into the palliative treatment concept”, “Practical advice on how to keep social functions (within and outside the family)” and “Desired equal participation in internal decision-making processes”.

The sub-theme “Practical advice on integrating private life into the palliative treatment concept” illustrated the necessity of developing individual palliative medical treatment concepts and the consideration of patient preferences. Within the MCA, patients wanted to decide for themselves how much support they adopt.

“It’s nice that they’re here (...) if there’s something really stupid, then I know I can get in touch with them. But as I said, I don’t necessarily have to make use of it [currently].” (P05)

For participants, to continue a normal life in which the disease is not predominant was also important.

“It has been discovered very late and we are trying to get the best out of this situation and we have to change things, that we bring our life together in the foreground and other things in the background.” (IC30).

The role of informal caregivers was expressed by the sub-theme “Practical advice on how to keep social functions [within and outside the family]”. Family cohesion and the support of patients were perceived as major tasks.

“I always think that four ears hear better than two, and that was very supportive for me that he [husband] [was there]. And that we talked it over at home.” (P01)

Informal caregivers also expressed their wish to play a supporting role, but leaving the main responsibility with the patient as long as possible.

“During the conversation I hold myself back and try to say a few things that my wife sometimes forgets to say (...) She is the patient and she should do it and I am not there to guide her. I just try to help a little bit with information and writing it down.” (IC30)

The sub-theme “Desired equal participation in internal decision-making processes” within the MCA shows a high degree of diversity. While for some patients the medical competence was the exclusive decision option, other patients liked to make the sole decision about the further therapeutic procedure themselves. A further group of patients saw the best way for treatment processes in shared decision-making, considering the recommendation of the service providers and their own acceptance.

“I said to the physician, ‘If this is what you suggest, this is what we do. I can’t say anything about it.’” (P12)

Participants also noticed that the tandem in the context of the MCs supported and encouraged the shared decision-making process. After explaining different treatment options, the patients could decide for themselves about the therapeutic path.

“The physician said, ‘You’re the boss, you decide, and we’re okay with it. How you decide, we’ll do it.’” (P02)

### Theme 3: manageability

The theme “manageability” comprised the sub-themes “Dealing with burdensome problems concerning the disease situation”, “Development of the social support network” and “Anticipatory coping advice in the context of the disease situation for palliative lung cancer patients”.

In the sub-theme “Dealing with burdensome problems concerning the disease situation”, the presence of a physician and a nurse led to psychological relief, acceptance of support, recognition and elimination of uncertainties and to the release of blockades.

“The openness (...) and the willingness to give you an answer to every question, (...) you feel taken seriously and there is understanding that you just have questions.” (IC01)

Resuming or maintaining existing routines and hobbies in everyday life, being around pets and taking walks led to activities that brought relief to the patient.

“Now I’ve started playing bridge again and we go to the theatre and concerts. So, we’re not at home moping around.” (P01)

Anticipatory coping advice in the context of the disease and the limited prognosis became important for palliative patients. For most participants, responding to and accompanying palliative medical measures such as palliative medical treatment on a palliative ward or in a hospice, drafting a living will and clarifying family matters were crucial.

“So just the subject of advance directive (...) I don’t want to do it alone, because it really affects me and takes me along, but I also know that if I don’t do it and it turns out to be something, then my husband cannot decide everything for me.” (P02)

Nevertheless, it was occasionally clear that the MCs were sometimes too extensive and felt to be too intense and challenging.

“The talks were very detailed, very good and sometimes they were annoying (…) Because there was just too much asking, and when you go through something like that for the first time, then I found it sometimes actually too much.” (Patient 02)

## Discussion

This study explored perspectives and experiences from lung cancer patients and informal caregivers to gain an understanding of the extent to which salutogenetic aspects were considered in the MCA for patients with a limited prognosis. Participants perceived the interprofessional tandem as an added value for patient care, in which SOC was strengthened. In particular, the presence and involvement of nurses were highly valued in the scheduled MCs and follow-up calls to enhance comprehensibility and manageability of the burdensome situation. Participants also used the MCs as a framework to discuss the topics of PA and ACP. Nevertheless, different needs of patients require an individual adaptation of the MCs to achieve a minimisation of the psychological burden.

For most patients and informal caregivers, the diagnosis of lung cancer comes suddenly and surprisingly, causing an exceptional psychological and emotional situation. At the same time, they receive a lot of healthcare information, including treatment options, further procedures and organisational aspects [12]. In this situation, the interprofessional tandem is perceived as a great gain for patient care and support. Within the context of the MCs, the nurse encourages a low-threshold entry into the MCs and thus contributes to a greater sense of well-being. By building a stable relationship to patients and informal caregivers, nurses not only ensure continuity of care [13] but also support in difficult communications [14].

A positive relation between SOC and quality of life has been shown in previous studies in lung cancer patients [15, 16]. Identifying patients with a lower SOC leads to individual and tailored care which accounts for patients’ resources. In general, the communication approach helps patients to activate their SOC, and choosing appropriate coping strategies [3] which early in the disease trajectory positively influences patients’ quality of life [17]. Regular contact and use of appropriate language by the physician-nurse team strengthen comprehensibility among patients and informal caregivers. Comprehensibility can be interpreted as the basis for coping with a life-threatening situation. Knowing about the situation (comprehensibility), accepting it (meaningfulness) and dealing responsibly with it (manageability) led to individual and specific actions in concordance with the limited prognosis and ACP.

The support that participants receive from the MCs has to be connected to PA which is individual and strongly related to the personal desire for information of patients and informal caregivers. Depending on the individual patient, the preference for receiving information varied greatly. Some lung cancer patients wanted to be fully informed by their healthcare professional about their condition and treatment options, including the prognosis, despite its limitations. In contrast, the full information was overwhelming and frightening for other patients, who therefore wanted only limited information [18]. Respecting patients’ autonomy, also in not wanting to know, enables patients to decide based on their individual values and preferences [19]. Furthermore, the offer of supportive communication about the prognosis allows patients to engage in ACP [20].

For most patients, informal caregivers and a normal daily routine are of major importance. By maintaining social contacts with family members and friends, the disease does not become the sole focus of life. Patients thus keep their social role which is a significant part of their quality of life [21]. As Harrop et al. in their study with lung cancer patients also observed, the sense of normality in maintaining daily routines supports strategies to cope with a challenging situation [22]. Healthcare professionals could improve the use of coping strategies by addressing these resources in their communication with patients and informal caregivers.

### Study limitations

Although participants did not provide new topics in the last conducted interviews, suggesting information saturation, other patients and informal caregivers might have given further insights which have not yet been considered. In particular, participants in our study emphasised mainly positive aspects and experiences which could imply a selection bias in the sample in our study. The patients were interviewed after having known the diagnosis with its limited prognosis for at least two months and maybe adapted to the situation in the meantime. Since a higher SOC is related to lower distress [4, 23], they seem to have found a way of coping with the situation.

Another limitation of our study is the consideration of patients’ and informal caregivers’ accounts as independent. Participants might have experienced the same situation differently. Still, we did not triangulate the interviews since the focus of the study was on a general perception of salutogenetic aspects, not on the completeness within the MCs or the congruence of patient and informal caregiver reports.

The purpose of this study required the researchers to focus on the core components of SOC and the Model of Communication to Integrate Early Palliative Care in Thoracic Oncology. This approach implied a potential neglect of other identified themes relevant for patient care. An additional confounder might be the selection of patients and informal caregivers receiving the MCA intervention; patients receiving standard care might have provided general insights in challenging communication. Nevertheless, considering patients at the centre of care within the MCA, a focus on their resources facilitates approaches to strengthen individual care and further develop the communication concept.

### Clinical implications

A structured communication approach with lung cancer patients and their informal caregivers can support them in coping with a challenging information. Addressing communication needs and enhancing patients’ SOC by pointing out personal and social resources of patients and informal caregivers are an important part of patient-professional communication.

## Conclusions

Salutogenetic aspects are relevant in healthcare communication with lung cancer patients with a limited prognosis. Supporting the patients with understanding, accepting and handling their situation also encourages patient empowerment and facilitates ACP. In this way, the high psychological burden often associated with a limited cancer prognosis can be counteracted, which in turn has a positive effect on patients’ and informal caregivers’ quality of life. Patients and caregivers highlighted the nurses’ role in salutogenic aspects. By strengthening interprofessional collaboration through the tandem-approach, these aspects are more likely to be integrated into oncological care.

## Data Availability

Available from the corresponding author on reasonable request.
